# Health economic assessment of Gd-EOB-DTPA MRI versus ECCM-MRI and multi-detector CT for diagnosis of hepatocellular carcinoma in China

**DOI:** 10.1371/journal.pone.0191095

**Published:** 2018-01-11

**Authors:** Xiaoning He, Jing Wu, Anke-Peggy Holtorf, Harald Rinde, Shuangshuang Xie, Wen Shen, Jiancun Hou, Xuehua Li, Ziping Li, Jiaming Lai, Yuting Wang, Lin Zhang, Jian Wang, Xuesong Li, Kuansheng Ma, Feng Ye, Han Ouyang, Hong Zhao

**Affiliations:** 1 Department of Health and Pharmacy Administration, School of Pharmaceutical Science and Technology, Tianjin University, Tianjin, China; 2 Health Outcomes Strategies, Basel, Switzerland; 3 Department of Radiology, Tianjin First Center Hospital, Tianjin, China; 4 Department of Hepatobiliary Surgery, Tianjin First Center Hospital, Tianjin, China; 5 Department of Radiology, The First Affiliated Hospital, Sun Yat-sen University, Guangzhou, China; 6 Department of Hepatobiliary Surgery, The First Affiliated Hospital, Sun Yat-sen University, Guangzhou, China; 7 Department of Radiology, Southwestern Hospital, Chongqing, China; 8 Department of Hepatobiliary Surgery, Southwestern Hospital, Chongqing, China; 9 Department of Radiology, Cancer Hospital Chinese Academy of Medical Sciences, Beijing, China; 10 Department of Hepatobiliary Surgery, Cancer Hospital Chinese Academy of Medical Sciences, Beijing, China; Yonsei University College of Medicine, REPUBLIC OF KOREA

## Abstract

Limited data exists in China on the comparative cost of gadolinium ethoxybenzyl diethylenetriamine magnetic resonance imaging (Gd-EOB-DTPA-MRI) with other imaging techniques. This study compared the total cost of Gd-EOB-DTPA-MRI with multidetector computed tomography (MDCT) and extracellular contrast media–enhanced MRI (ECCM-MRI) as initial imaging procedures in patients with suspected hepatocellular carcinoma (HCC). We developed a decision-tree model on the basis of the Chinese clinical guidelines for HCC, which was validated by clinical experts from China. The model compared the diagnostic accuracy and costs of alternative initial imaging procedures. Compared with MDCT and ECCM-MRI, Gd-EOB-DTPA-MRI imaging was associated with higher rates of diagnostic accuracy, i.e. higher proportions of true positives (TP) and true negatives (TN) with lower false positives (FP). Total diagnosis and treatment cost per patient after the initial Gd-EOB-DTPA-MRI evaluation was similar to MDCT (¥30,360 vs. ¥30,803) and lower than that reported with ECCM-MRI (¥30,360 vs. ¥31,465). Lower treatment cost after initial Gd-EOB-DTPA-MRI was driven by reduced utilization of confirmatory diagnostic procedures and unnecessary treatments. The findings reported that Gd-EOB-DTPA-MRI offered higher diagnostic accuracy compared with MDCT and ECCM-MRI at a comparable cost, which indicates Gd-EOB-DTPA-MRI could be the preferred initial imaging procedure for the diagnosis of HCC in China.

## Introduction

GLOBOCAN 2012 statistics lists liver cancer as a disease of the developing nations, the sixth most common cancer and the second leading cause of cancer deaths worldwide. Of the 782,500 new reported cases and 745,500 deaths worldwide, majority of the new cases and deaths were reported in less developed nations (648,000 and 622,000, respectively) [1,2]. China alone accounted for >50% of the total cases and deaths [1]. Hepatocellular carcinoma (HCC) comprises of 70% to 90% of the total liver cancers; is associated with high recurrence, poor prognosis and short survival time [3–5]. HCC is further associated with high cost of treatment [6–7].

HCC is mostly diagnosed at incurable stages, which severely affects the prognosis and decreases the survival to <1 year [8]. Therefore, early diagnosis and effective treatment approach should be followed in order to increase the survival rate and long-term survival for patients with HCC. However, early diagnosis of HCC is faced with challenge of late appearance of symptoms, variability of imaging features of HCC lesions and high prevalence of benign lesions [8–10]. Contrast media–enhanced multidetector computed tomography (MDCT), extracellular contrast media–enhanced magnetic resonance imaging (ECCM-MRI) and gadoxetate dimeglumine (Gd-EOB-DTPA) enhanced MRI are among the most widely used imaging techniques for HCC diagnosis [11]. However, lesions <2 cm in size (approximately 40%–75% of HCC lesions) require 2 serial imaging diagnoses, instead of direct confirmatory imaging with MDCT or ECCM-MRI [12–15]. This leads to a delay in HCC treatment and a serious deterioration in patient’s prognosis and outcome.

Gd-EOB-DTPA (Primovist, Bayer Healthcare, Berlin, Germany), a liver-specific MRI contrast media for detecting and characterizing focal liver lesions [16], was approved in China in the year 2010. The contrast agent enables dynamic perfusion imaging and allows evaluation of delayed hepatocyte uptake and biliary excretion [17]. By combining the hepatobiliary phase and dynamic MRI, this contrast agent can be used to differentiate malignant tumors from benign lesions and simultaneously provide information on the dynamic and specific effects over short periods of time [18,19]. Previously published clinical data have also confirmed higher accuracy of lesion detection and characterization with Gd-EOB-DTPA–MRI compared with MDCT and ECCM-MRI [19,20].

Along with greater diagnostic accuracy, Gd-EOB-DTPA-MRI is also associated with higher initial diagnosis cost in HCC patients [21]. Health economic evaluations of Gd-EOB-FTPA-MRI compared with MDCT and ECCM-MRI have been conducted in Europe, South Korea, and Thailand previously. These studies demonstrated a decrease in the number of confirmatory diagnostic procedures and surgeries after initial Gd-EOB-DTPA-MRI diagnosis. Based on the findings, Gd-EOB-DTPA-MRI was considered to be associated with cost savings [22,23]. To date, no health economic evaluation comparing Gd-EOB-DTPA-MRI with other imaging techniques has been conducted in China. Keeping in view the Chinese health care perspective, this study compared the diagnostic accuracy (by measuring sensitivity and specificity) and total costs associated with Gd-EOB-DTPA-MRI as an initial imaging procedure with MDCT and ECCM-MRI in suspected HCC patients in China.

## Research design and methods

### Preliminary model

A preliminary decision-tree model, which simulates the clinical pathway of patients with suspected HCC from the initial imaging until the treatment procedures, was developed using the current Chinese clinical guideline for diagnosis and therapy of HCC [5], and then validated by an expert panel with 14 experienced radiologists and surgeons in China.

### Patient characteristics

The population included in the health economic model consisted of suspected early to intermediate-stage HCC patients with one of the following characteristics: (i) lesion(s) of size >1 cm or growing lesion(s) of size <1 cm during monitoring detected by ultrasound; (ii) lesions of size <1cm detected by ultrasound and a history of HBV infection; (iii) A-fetoprotein (AFP)>400 μg/L or in case of progressively increasing AFP during monitoring; (iv) AFP>100 μg/L and a history of HBV infection. Patients with liver disease status of Child-Pugh Class C or worse, known invasive tumor pattern, or known extrahepatic metastasis were excluded considering they were at advanced stages.

### Input data collection

#### Literature review

Input data were collected either from the published literature or expert interviews. A literature review was conducted using PubMed to extract data on the performance of the three imaging procedures at specific branches. When several data sources were available, the average was calculated, S1 Table [10,24–29]. Performance of the second or third imaging procedures, which could not be sourced from the published literature were assumed to be equal to those for lesions <2 cm, S2 Table [24,25,27].

#### Delphi panel

Due to non-availability of published HCC prevalence data, a nationwide survey, using a Delphi Panel approach, was conducted among HCC experts to determine the true HCC prevalence among patients at high risk in 2015. For the purpose of conducting this survey, a total of 26 highly experienced radiologists or liver surgeons were recruited from 12 hospitals located in Beijing, Shanghai, Guangzhou, Shenzhen, Nanjing, Zhengzhou, Chongqing, and Suzhou. Participating experts were asked to estimate the lowest, most likely, and the highest true HCC prevalence according to their experience through several rounds of data collection. In each subsequent round, the experts received an anonymous summary of the experts’ estimates from the last round, and were encouraged to reconsider and revise their earlier estimates [30].

#### Final model structure

**Fig 1:**
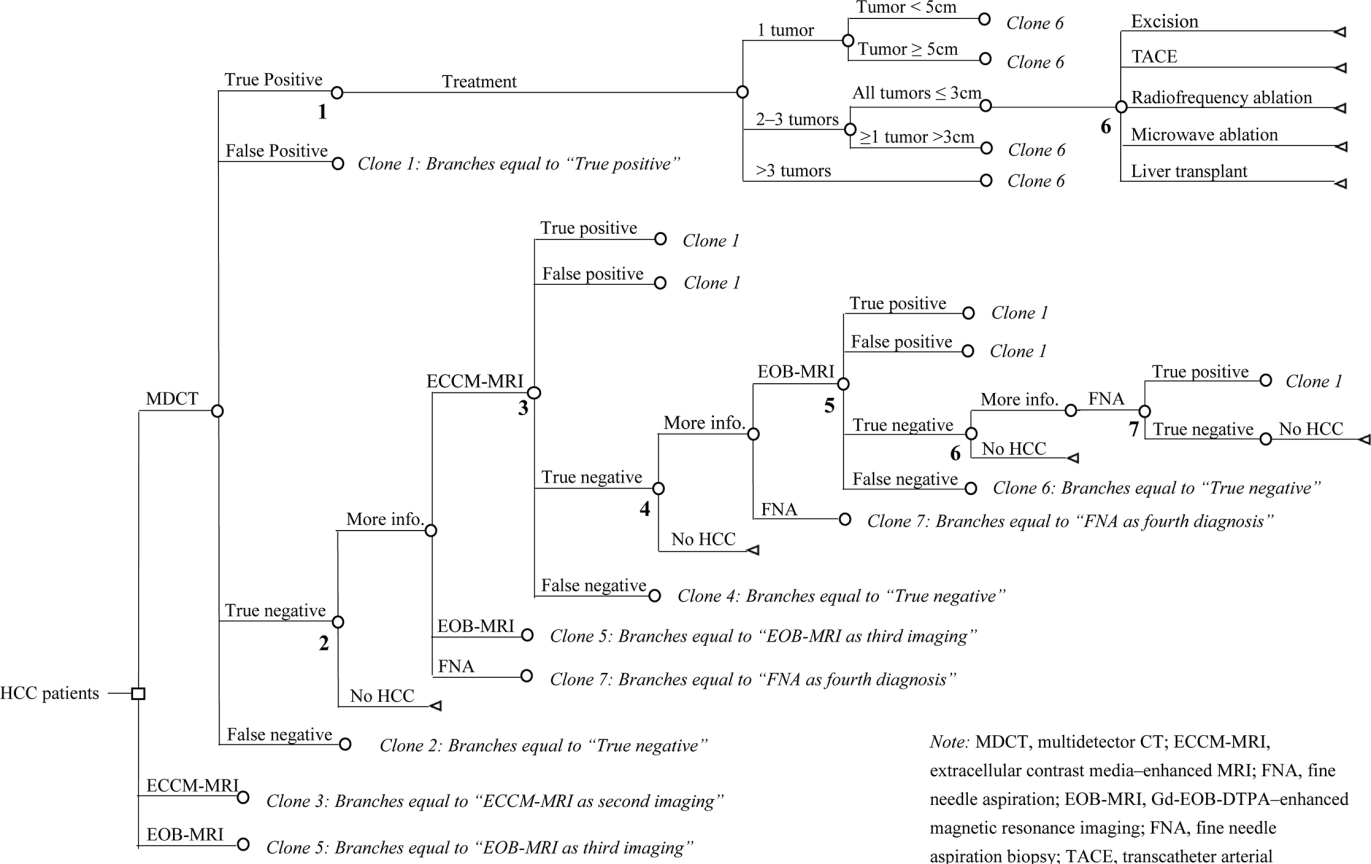
Model structure for the decision-tree used in the economic evaluation. *Note*: MDCT, multidetector computed tomography; ECCM-MRI, Extracellular contrast media–enhanced MRI; GD-EOB-DTPA-MRI, Gd-EOB-DTPA–enhanced magnetic resonance imaging.

In the final 7-step decision-tree model, the patients were initiated with imaging using MDCT, ECCM-MRI, or Gd-EOB-DTPA-MRI, and were confirmed with or without HCC diagnosis. Treatment was instituted in patients after the confirmation of HCC diagnosis (Fig 1). The results of imaging were reported as either positive (including true positive (TP) and false positive (FP)), which implies confirmed HCC; or negative (both true negative (TN) and false negative (FN)), which implied exclusion of HCC and the undetermined lesions. Patients confirmed with HCC underwent treatment; whereas the patients with undetermined lesion(s) underwent further diagnostic procedures including ECCM-MRI, Gd-EOB-DTPA-MRI, and/or fine needle aspiration (FNA) biopsy, until confirmation or exclusion. Patients confirmed without HCC were excluded from the model directly. Diagnosis was performed using Chinese clinical guideline for diagnosis and therapy of HCC.

Patients confirmed with HCC were divided into groups based on the number and size of the lesions present. The groups were (i) 1 tumor, <5 cm; (ii) 1 tumor, ≥5 cm; (iii) 2–3 tumors, all ≤3 cm; (iv) 2–3 tumors, at least 1 tumor >3 cm; (v) ≥4 tumors. Alternative therapeutic procedures including excision, transcatheter arterial chemoembolization (TACE), radiofrequency ablation (RFA), microwave ablation, and liver transplant were considered, with various probabilities depending on the number and size of the lesions. Chemotherapy, radiotherapy, molecular targeted treatment, and systematic therapy for patients with HCC at advanced stages were not considered.

The experts were also interviewed face-to-face to determine the transition probabilities and costs in diagnostic and treatment pathways, as these data could not be sourced from the published literature. The data were collected by interviewing four pairs of experts from four leading tertiary hospitals located in Beijing, Tianjin, Chongqing, and Guangzhou. Each consisted of one radiologist and one surgeon. All input data were collected via a three-round Delphi approach and then averaged across four pairs of experts. Estimated probabilities included: (i) probabilities for needing further diagnostic procedures or confirmed exclusion of HCC among patients at each imaging; (ii) probabilities for each of the specific possible subsequent diagnostic procedures at each step of the decision-tree; (iii) distribution probability of the number and size for lesions with a confirmed HCC diagnosis; and (iv) probabilities of receiving different treatment procedures under different tumor groups.

#### Sensitivity analysis

A series of one-way sensitivity analyses were performed to determine the robustness of the model and explore the impact of uncertainty around the input data on the model results. Parameters for sensitivity analysis included true HCC prevalence, all transition probabilities and costs. A variation of ±10% around the base case value was tested for each parameter. For the diagnostic performance, the highest and lowest values published in the literature were used as the upper and lower limits in the sensitivity analyses. Tornado graphs were used to present the key drivers of model results by order of importance. These analyses assess the sensitivity of the models to the parameter estimates reported by the clinicians, and estimate the direct impact of treatment pathway characteristics on the cost of diagnosis with Gd-EOB-DTPA-MRI.

The requirement for ethical approval was waivered for this study as none of the patients were directly involved and there was no direct impact on patient care. In addition, all the panelists were fully informed and provided their consent for participating in the Delphi process.

## Results

### Estimated inputs

The experts included in the survey were asked for the diagnostic technique followed, while the specificity and sensitivity data were taken from the literature. Thus, the final input data of diagnostic performance for imaging procedures was generated from the published literature or expert interviews, Table 1. As per the input data, Gd-EOB-DTPA-MRI had higher diagnostic sensitivity and specificity when compared with ECCM-MRI and MDCT at both the initial and subsequent imaging. The sensitivity and specificity of FNA biopsy were assumed to be 100%. In patients with negative results, the estimated input probability of requiring further information in non-HCC patients was least in patients diagnosed with initial CT followed by Gd-EOB-DTPA-MRI (29.2%) and Gd-EOB-DTPA-MRI as initial screening (31.8%), Table 2. All the patients (100%) undergoing Gd-EOB-DTPA-MRI as initial imaging procedure or as follow-up to initial CT or ECCM-MRI had the probability of undergoing FNA in case further information was needed at each imaging, Table 3. At the time of inclusion into the health economic model, majority (47.1%) of patients with confirmed HCC had 1 tumor of <5 cm, 25.3% patients had 1 tumor of ≥5cm. Incidence rate of 2–3 tumors of either ≤3cm or >3cm was 8.2% and 6.4%, respectively (S1 Fig).

**Table 1:** Input probability for diagnostic accuracy for initial and subsequent imaging procedures.

Imaging procedures	Initial imaging		Subsequent imaging	
	Sensitivity	Specificity	Sensitivity	Specificity
MDCT	73.4%	91.4%	70.0%	83.0%
ECCM-MRI	79.7%	87.3%	72.0%	78.9%
Gd-EOB-DTPA-MRI	92.3%	95.3%	87.0%	93.0%

**Table 2:** Input probabilities for needing further information on non-HCC confirmed among patients with negative results at each imaging.

Diagnostic node	Further info. needed	No HCC
MDCT as initial imaging	61.4%	38.6%
ECCM-MRI as initial imaging	51.6%	48.4%
Gd-EOB-DTPA-MRI as initial imaging	31.8%	68.2%
Initial CT and follow-up on ECCM-MRI	70.6%	29.4%
Initial CT and follow-up on Gd-EOB-DTPA-MRI	29.2%	70.8%
Initial ECCM-MRI and follow-up on Gd-EOB-DTPA-MRI	42.7%	57.3%
Initial CT, follow-up on ECCM-MRI, then Gd-EOB-DTPA-MRI	42.9%	57.1%

**Table 3:** Input probabilities for subsequent diagnostic procedures arranged when further information was needed at each imaging.

Diagnostic node	ECCM-MRI	Gd-EOB-DTPA-MRI	FNA
MDCT as initial imaging	25.0%	65.8%	9.2%
ECCM-MRI as initial imaging	—	79.3%	20.8%
Gd-EOB-DTPA-MRI as initial imaging	—	—	100%
Initial CT and follow-up on ECCM-MRI	—	52.5%	47.5%
Initial CT and follow-up on Gd-EOB-DTPA-MRI	—	—	100%
Initial ECCM-MRI and follow-up on Gd-EOB-DTPA-MRI	—	—	100%
Initial CT, follow-up on ECCM-MRI, then Gd-EOB-DTPA-MRI	—	—	100%

Costs input were estimated from the Chinese health care perspective. Unit costs of MDCT, ECCM-MRI, Gd-EOB-DTPA-MRI, and FNA biopsy were collected and averaged. Treatment costs during one hospitalization, including the costs for excision, TACE, RFA, microwave ablation, or liver transplant were also estimated by the experts, S3 Table.

Final HCC true prevalence was calculated as the average rate of the lowest, most likely, and highest estimated rates using the Delphi approach. The consensus was reached after three rounds of data collection with a final estimated true HCC prevalence of 47.0%. The detailed survey data is presented in S4 Table. S1 Fig presents the overall distribution probability of patients with confirmed HCC as per the tumor numbers and sizes. S5 Table shows the calculated process of the weighted treatment cost; the treatment cost per patient with confirmed HCC was calculated to be ¥57,998. Excision was the major treatment modality for all the tumor groups, except patients with >3 tumors, in whom TACE was the major treatment choice.

### Diagnostic accuracy with Gd-EOB-DTPA-MRI is higher than ECCM-MRI and MDCT

**Fig 2:**
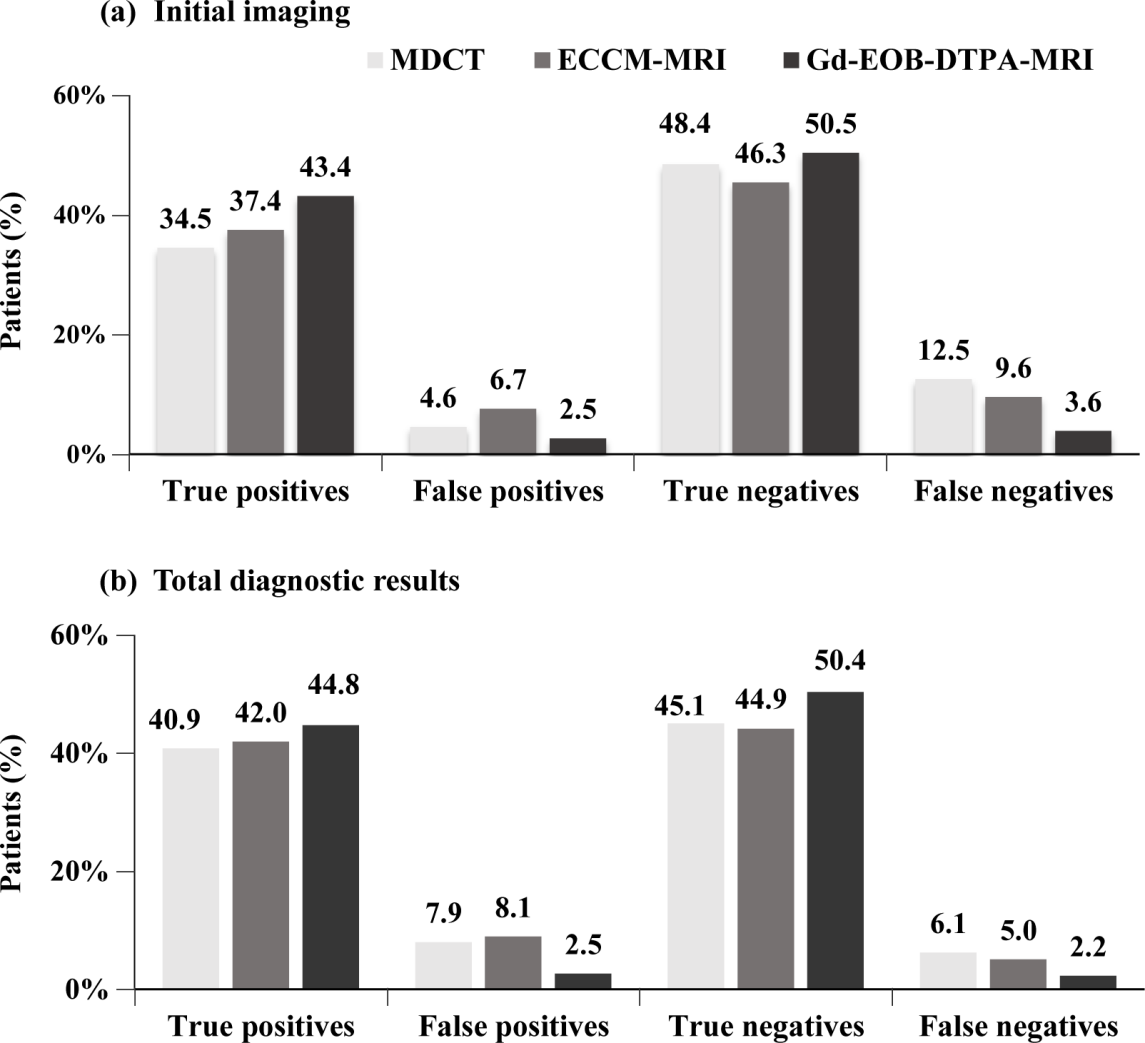
Distribution of patients after initial imaging and all diagnostics. *Note*: MDCT, multidetector computed tomography; ECCM-MRI, Extracellular contrast media–enhanced MRI; GD-EOB-DTPA-MRI, Gd-EOB-DTPA–enhanced magnetic resonance imaging.

During the initial imaging procedure for HCC, the proportion of TP patients for the Gd-EOB-DTPA-MRI group was higher compared to ECCM-MRI and MDCT (43.4% vs. 37.4% and 34.5%). FP and FN proportion with Gd-EOB-DTPA-MRI during the initial imaging were reported lower in comparison to ECCM-MRI and MDCT (FP: 2.5% vs. 6.7% and 4.6%; FN: 3.6% vs. 9.6% and 12.5%, Fig 2A). No additional confirmatory imaging was required in Gd-EOB-DTPA-MRI group, whereas 34.0% in the MDCT and 22.8% in the ECCM-MRI group required additional confirmatory imaging. Patients in the Gd-EOB-DTPA-MRI group had no additional confirmatory imaging tests, but underwent FNA.

Higher proportion of TPs with Gd-EOB-DTPA-MRI was also reported at the end of the diagnostic procedure compared to ECCM-MRI and MDCT (44.8% vs. 42.0% and 40.9%). Proportion of FPs at end of diagnosis using ECCM-MRI and MDCT were higher compared with initial diagnosis (ECCM-MRI: 8.1% vs. 6.7%; MDCT: 7.9% vs. 4.6%). Application of additional confirmatory diagnostic tests lowered the incidence rates of FNs in the MDCT (12.5% to 6.1%), ECCM-MRI (9.6% to 5.0%) and Gd-EOB-DTPA-MRI (3.6% to 2.2%), Fig 2B.

### Overall treatment cost after diagnosis with Gd-EOB-DTPA-MRI, ECCM-MRI and MDCT

In China, a diagnosis of HCC using Gd-EOB-DTPA-MRI incurs a higher cost for the patient (¥2,549) compared with MDCT (¥1,528), ECCM-MRI (¥1,558) and fine needle aspiration (FNA, ¥1,981). The treatment cost is the highest for liver transplant (¥261,032), excision (¥60,832), RFA (¥31,750), microwave ablation (¥29,500) and TACE (¥25,682). The weighted per patient cost for HCC treatment is reported to be ¥57,998, S5 Table.

**Fig 3:**
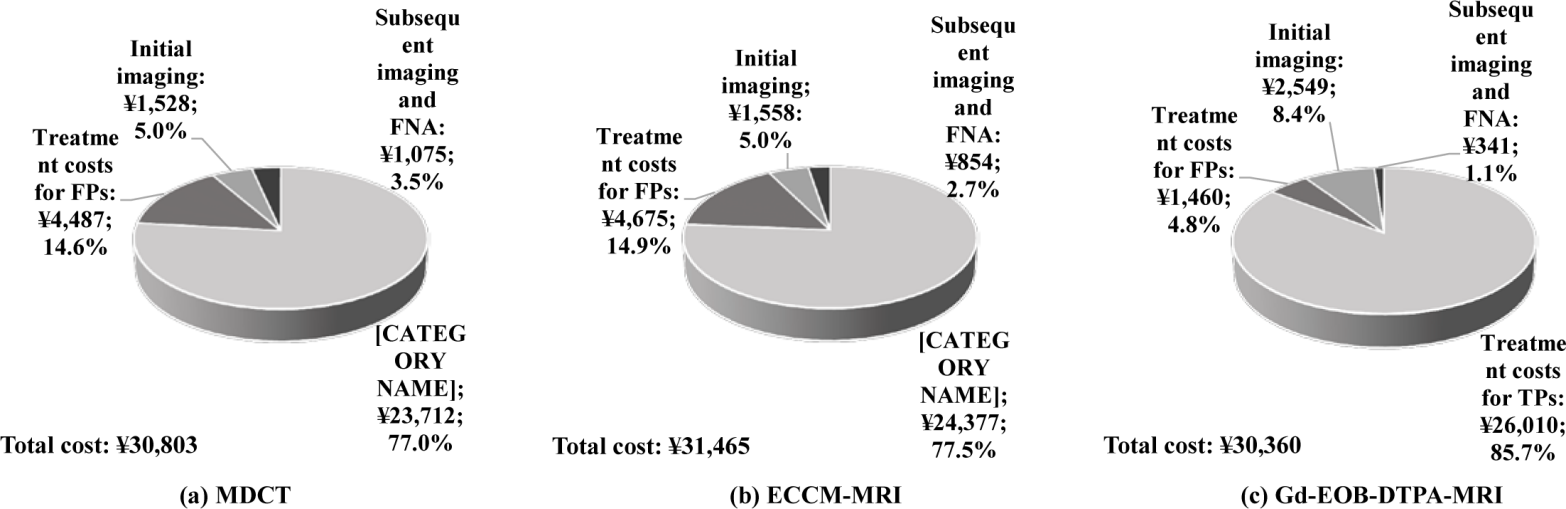
Total costs of diagnostic and treatment procedures depending on initial imaging. *Note*: MDCT, multidetector computed tomography; ECCM-MRI, Extracellular contrast media–enhanced MRI; GD-EOB-DTPA-MRI, Gd-EOB-DTPA–enhanced magnetic resonance imaging.

Despite a higher cost of initial diagnosis with Gd-EOB-DTPA-MRI the total cost of diagnosis and treatment procedures per patient with Gd-EOB-DTPA-MRI was comparatively lower than that of ECCM-MRI (¥30,360 vs. ¥31,465) and MDCT (¥30,360 vs. ¥30,803), Fig 3. In case of TPs, the total per patient cost mainly comprised of cost for treatment procedure in all the groups. In the patients diagnosed with Gd-EOB-DTPA-MRI, the cost for TP treatment procedure was 85.7% of the total treatment cost. In the ECCM-MRI and MDCT diagnosed patients, TP treatment cost procedure was 77.5% and 77.0%, respectively. In case of the FP diagnosis, treatment cost was the highest in the ECCM-MRI group (14.9%, ¥4,675), followed by MDCT (14.6%, ¥4,487) and Gd-EOB-DTPA-MRI (4.8%, ¥1,460). Costs for subsequent imaging procedures and FNA biopsy constituted 1.1% in the Gd-EOB-DTPA-MRI arm (¥341), 2.7% in the ECCM-MRI arm (¥854) and 3.5% in the MDCT arm (¥1,075). Fig 3 presents the overall diagnosis and treatment cost distribution in all the 3 groups.

### Sensitivity analysis

**Fig 4:**
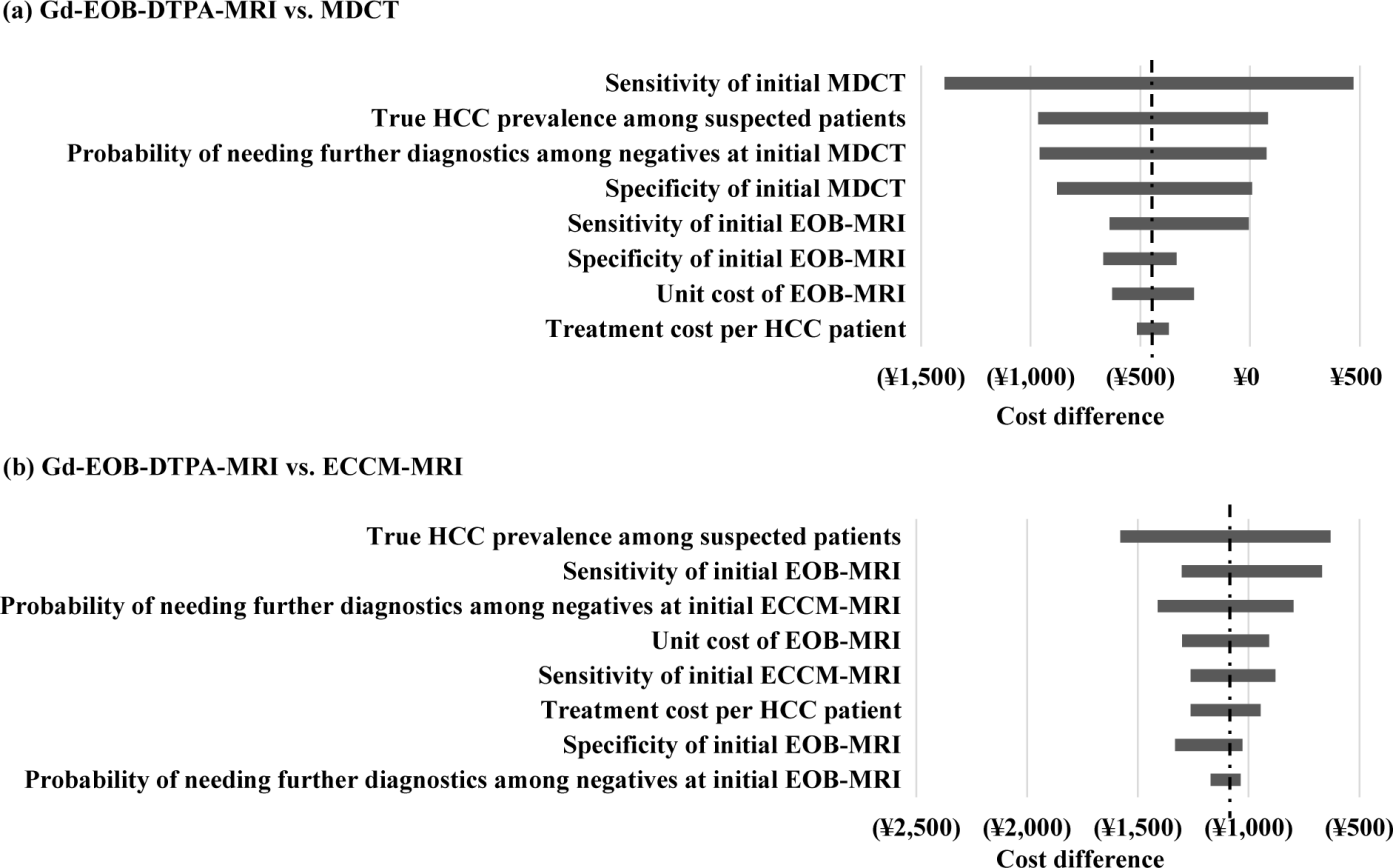
One-way sensitivity analyses. *Note*: MDCT, multidetector computed tomography; ECCM-MRI, Extracellular contrast media–enhanced MRI; GD-EOB-DTPA-MRI, Gd-EOB-DTPA–enhanced magnetic resonance imaging.

To further evaluate the cost difference between Gd-EOB-DTPA-MRI and the other two diagnostic procedures, one-way sensitivity analyses were performed. At base case, cost of Gd-EOB-DTPA-MRI was -¥442 compared with MDCT (¥30,360 vs. ¥30,803). Increase in sensitivity of MDCT to 81.0% (from base case of 73.4%) and decrease in true HCC prevalence (10% from base case of 47.0%) were responsible for causing the greatest impact on the cost difference between Gd-EOB-DTPA-MRI and MDCT (-¥1,393 and -¥966, respectively) compared with the base case. On lowering the sensitivity of MDCT to 66.6%, cost of initial MDCT was slightly lower compared with Gd-EOB-DTPA-MRI (difference: ¥471), since more patients with HCC were missed and not treated, which led to overall saving. Cost saving with initial MDCT was also observed when the true HCC prevalence was increased by 10% (difference: ¥81). The probability of undetermined lesions among negative results at initial imaging and the specificity of initial MDCT also had a moderate effect on the model results (S6 Table). Fig 4A presents the impact of key variables on the cost incurred with Gd-EOB-DTPA-MRI and MDCT. Among the key variables sensitivity of initial MDCT had the greatest impact and treatment cost per patient had the least impact on the cost difference among the two groups.

At base case, the cost of diagnosis with Gd-EOB-DTPA-MRI was lower than those reported for ECCM-MRI (difference: -¥1,104). Increase in true HCC prevalence by 10% lowered the cost difference between the two diagnostic procedures from the base case value (from -¥1,104 to -¥630); whereas decrease in true prevalence of HCC further increased the cost difference between Gd-EOB-DTPA-MRI and ECCM-MRI (from -¥1,104 to -¥1,579). All the other variables impacting the cost difference ranged from -¥669 to -¥1,411 (S7 Table). Fig 4B compares the total cost between Gd-EOB-DTPA-MRI and ECCM-MRI in terms of the key variables impacting the cost of diagnostic procedure and revealed that true HCC prevalence had the greatest impact on the cost and probability of needing further diagnostics among negatives at initial Gd-EOB-DTPA-MRI had the least impact on the cost.

## Discussion

As China has a large patient pool of HCC; and thousands of new cases are added to the already existing patient population^1^, therefore diagnosis becomes a vital step for early detection and effective treatment. The most commonly used imaging techniques, MDCT and ECCM-MRI, however cannot completely detect HCC lesions <2cm in size. Furthermore, cost of imaging procedure also plays a role in selection of the procedure [31]. As per our knowledge, this was the first health economic study comparing the diagnostic accuracy and total costs of using MDCT, ECCM-MRI, and Gd-EOB-DTPA-MRI as initial imaging procedures for patients with suspected HCC in China.

Multiple studies have reported higher diagnostic accuracy of initial Gd-EOB-DTPA-MRI compared with other imaging techniques; which indicates that more number of patients are correctly diagnosed using Gd-EOB-DTPA-MRI and require less additional confirmatory procedures. Alaboudy et al. compared the utility of Gd-EOB-DTPA-MRI compared with contrast-enhanced CT and enhanced ultrasound in 32 patients undergoing surgical resection. Compared with CT and enhanced ultrasound, Gd-EOB-DTPA-MRI had higher sensitivity (86% vs. 74% and 72%) showing the usefulness of Gd-EOB-DTPA-MRI for HCC diagnosis [32]. Furthermore, Inoue et al. reported significantly higher detection ability of Gd-EOB-DTPA-MRI compared with MDCT in detecting hypervascular lesions <2cm (P = 0.048) and hypovascular lesions (P = 0.001) in Japanese patients with HCC [33]. Significantly higher overall detection rate (P<0.05) and sensitivity (P<0.05) were reported with Gd-EOB-DTPA-MRI compared with MDCT in a single-center study in Germany [34]. Multiple studies showed higher detection of HCC lesions with MRI (TP) and lower inclusion of non-HCC (FN) after initial diagnosis with ECCM-MRI and MDCT [14,35,36]. Consistent with the previous findings, Gd-EOB-DTPA-MRI reported higher proportion of cases of TP and TN diagnosis compared with alternative imaging procedures at initial imaging and at the end of the diagnosis, along with showing lower need for additional confirmatory procedures. Similarly, the proportion of patients diagnosed as FP and FN was lower with Gd-EOB-DTPA-MRI. Lower rate of FP led to lower unnecessary diagnostic procedures or therapeutic interventions for the patients. Furthermore, lower proportion of patients diagnosed as FN could result in a considerable delay in therapy and potentially fatal consequences for the patient. This was of lower probability in our study due to lower proportion of patients diagnosed as FN after Gd-EOB-DTPA-MRI compared with MDCT and ECCM-MRI. Since the present data was sourced from a group of highly experienced specialists in China, the results of this study can be expected to portray the best-case scenario, as diagnostic performance depends on the experience and routine of the radiologist [37–40].

Several health economic evaluations of Gd-EOB-FTPA-MRI and alternative imaging procedures have been conducted previously. A model with European patients (Italy, Germany and Sweden) with suspected colorectal liver metastases reported that Gd-EOB-DTPA-MRI as initial diagnosis resulted in lower rate of further imaging required (8.6%) compared with ECCM-MRI (18.5%) and MDCT (23.5%), which translated to lowered intraoperative treatment costs and hence, supported the cost savings induced by using Gd-EOB-FTPA-MRI compared with the alternatives in Sweden. Gd-EOB-DTPA-MRI only reported cost savings compared with ECCM-MRI in Italy and Germany [22]. Similar cost evaluation model used in the multinational VALUE trial (conducted in Switzerland, Sweden, Austria, South Korea and Thailand) also reported less requirement of subsequent imaging and similar diagnostic costs of Gd-EOB-DTPA-MRI compared with alternative diagnoses. These findings supported the use of Gd-EOB-DTPA-MRI as recommended initial diagnostic procedure [41]. A similar health economic model was employed by radiologists and clinical experts to compare the costs of the diagnostic procedures for HCC patients in Korea and Thailand. The results from the model showed higher diagnostic accuracy with Gd-EOB-FTPA-MRI which led to overall savings in both the countries compared with the alternate procedures [23]. In China, the current standard-of-care diagnostic process is usage of MDCT or ECCM-MRI initially, as the cost of diagnosis is covered by the Chinese basic medical insurance. Usage of Gd-EOB-DTPA-MRI is either second or third line in China because the expenses for the procedure are incurred by the patients [42]. The cost evaluation in our study was consistent with the previous studies; and total cost (diagnostic and treatment cost) after using Gd-EOB-DTPA-MRI as the first diagnostic procedure was similar (vs. MDCT: ¥30,360 vs. ¥30,803) or lower (vs. ECCM-MRI: ¥30,360 vs. ¥31,465) due to the reduced utilization of subsequent diagnostic procedures, lower number of unnecessary treatment procedures for FPs and correction of the majority of FNs during subsequent imaging, usually by Gd-EOB-DTPA-MRI. These observations were made despite higher acquisition costs of Gd-EOB-DTPA-MRI, and support the usage of Gd-EOB-DTPA-MRI as first-line imaging procedure in China.

Around 15% of the total costs were used for unnecessary treatment among patients with FPs results in the initial MDCT and ECCM-MRI arms, which not only meant wastage of time and money but were associated with avoidable suffering and pain for the patients. We ignored the treatment costs for FNs due to the defined scope of the model. Hence, the overall benefit of using Gd-EOB-DTPA-MRI as the first imaging procedure was most likely underestimated. This would explain the effect seen in the one-way sensitivity analysis regarding the diagnostic sensitivity of initial MDCT: the lower the sensitivity of MDCT, lower the total cost it incurred compared with Gd-EOB-DTPA-MRI because more patients with HCC were declared as HCC negative and, thus, missed in the further cost calculation. Due to the lack of published input data, most of the data were estimated from a limited number of interviews with clinical expert panels, which were not as strong as the evidence from clinical trials. All of the participating experts were recruited from leading tertiary hospitals and had long clinical experience with the diagnosis and treatment pathway of HCC. Sensitivity analyses also confirmed the model robustness and consistency of the results. Among all the parameters, HCC prevalence among the starting population was the most sensitive input factor of the model results. The cost difference between Gd-EOB-DTPA-MRI and MDCT increased by ¥111 with each 1% increase in the prevalence of HCC in the starting population over the base case HCC prevalence of 47%.

Our study had a few limitations. First, the model was a simplified reflection of the clinical complexity in real life. Only key clinical pathways and outcomes were mapped in the model. The model simulation was simplified further by making some key assumptions such as the 100% of FNA diagnostic performance, which may not be true in clinical practice. Second, the same weighted average treatment cost was calculated for each patient with confirmed HCC regardless of the heterogeneity in tumor type and specific treatment received. Third, this health economic model study only estimated the direct medical costs without considering the patients’ productivity loss and quality of life. Fourth, as the data were estimated from experts’ interviews and not from clinical studies, the results should be interpreted with caution. Therefore, further studies should be conducted to cover these aspects and reveal a more complete picture.

## Conclusions

In summary, although Gd-EOB-DTPA-MRI costs higher compared with MDCT and ECCM-MRI, initial diagnosis using Gd-EOB-DTPA-MRI had overall similar cost as that of MDCT and lower cost compared with ECCM-MRI in Chinese patients with suspected HCC. The decreased costs were attributed to reduced subsequent diagnostic procedures, unnecessary treatments, and potential delays for confirmed diagnosis. Therefore, Gd-EOB-DTPA-MRI might be used as first-line diagnosis of HCC and other tumor types in China.

## Supporting information

S1 Fig Distribution probabilities of tumor number and size for patients with confirmed HCC. (TIF)

S1 Table Diagnostic performance retrieved from literature for initial imaging procedures. (DOCX)

S2 Table Diagnostic performance retrieved from literature for subsequent (second or third) imaging procedures. (DOCX)

S3 Table Costs of diagnostic and treatment procedures in China. (DOCX)

S4 Table Final true prevalence. (DOCX)

S5 Table Weighted treatment costs determined by distributions of tumor groups and treatment procedures. (DOCX)

S6 Table One-way sensitivity analysis of cost differences between Gd-EOB-DTPA-MRI and MDCT. (DOCX)

S7 Table One-way sensitivity analysis of cost differences between Gd-EOB-DTPA-MRI and ECCM-MRI. (DOCX)
