# Supplementary material for: Health economic assessment of Gd-EOB-DTPA MRI versus ECCM-MRI and multi-detector CT for diagnosis of hepatocellular carcinoma in China
Source: PLoS One. 2018 Jan 11;13(1):e0191095. doi: 10.1371/journal.pone.0191095 (PMC5764342; doi:10.1371/journal.pone.0191095)
Supplement: S1 Table — (DOCX) [file pone.0191095.s002.docx]

**S1 Table Diagnostic performance retrieved from literature for initial imaging procedures**

| **Reference** | **Sensitivity** | **Specificity** | **Comment** | **Number of studies included** |
| --- | --- | --- | --- | --- |
| **CT Initial imaging** | | |  |  |
| Chen 2013 [25] | 81.0% | 93.0% | MRI with liver-specific contrast agents; all sizes | Sensitivity: 15; specificity: 5 |
| Lee 2015 [24] | 68.0% |  | Per-lesion basis, 11 studies | Sensitivity: 11 |
| Chou 2014 [27] | 78.4% | 89.8% | Meta-analysis 241 publications (AHRQ); overall performance | Sensitivity: 9; specificity: 4 |
| Chou 2014 [27] | 66.0% |  | Direct (within-study) comparisons | Sensitivity: 3 |
| **Mean** | **73.4%** | **91.4%** |  |  |
|  |  |  |  |  |
| **MRI Initial imaging** | | |  |  |
| Lee 2015 [24] | 80.0% |  | Per-lesion basis | Sensitivity: 11 |
| Chou 2014 [27] | 81.0% | 85.7% | Meta-analysis 241 publications (AHRQ); overall performance | Sensitivity: 11; specificity: 6 |
| Chou 2014 [27] | 78.0% |  | Direct (within-study) comparisons | Sensitivity: 3 |
| Nishie 2017 [28] |  | 89.0% |  |  |
| **Mean** | **79.7%** | **87.3%** |  |  |
|  |  |  |  |  |
| **Gd-EOB-DTPA-MRI Initial imaging** | | |  |  |
| Chen 2013 [25] | 91.0% | 95.0% | MRI with liver-specific contrast agents; all sizes | Sensitivity: 15; specificity: 5 |
| Liu 2013 [10] | 91.0% | 95.0% | Gd-EOB-DTPA enhanced MRI; overall HCC | Sensitivity: 10; specificity: 10 |
| Lei 2014 [26] | 92.0% | 95.0% | 1,578 lesions | Sensitivity: 13; specificity: 13 |
| Ye 2015 [29] | 95.0% | 96.0% | Per-lesion analysis | 9 study meta-analysis |
| **Mean** | **92.3%** | **95.3%** |  |  |
