# Supplementary material for: Health economic assessment of Gd-EOB-DTPA MRI versus ECCM-MRI and multi-detector CT for diagnosis of hepatocellular carcinoma in China
Source: PLoS One. 2018 Jan 11;13(1):e0191095. doi: 10.1371/journal.pone.0191095 (PMC5764342; doi:10.1371/journal.pone.0191095)
Supplement: S2 Table — (DOCX) [file pone.0191095.s003.docx]

**S2 Table Diagnostic performance retrieved from literature for subsequent (second or third) imaging procedures**

| **Reference** | **Sensitivity** | | **Specificity** | | **Comment** | **Number of studies included** |
| --- | --- | --- | --- | --- | --- | --- |
| **CT subsequent imaging** | | | | |  |  |
| Chen 2013 [25] | | 73.0% | | 83.0% | MRI with liver-specific contrast agents; size <20 mm | Sensitivity: 15;  specificity: 5 |
| Chou 2014 [27] | | 63.0% | |  | < 2 cm |  |
| Chou 2014 [27] | | 74.0% | |  | Lesions, 1–2 cm |  |
| **Mean** | **70.0%** | | | **83.0%** |  |  |
|  |  | | |  |  |  |
| **MRI subsequent imaging** | | | | |  |  |
| Chen 2015 [24] | |  |  | |  |  |
| Chou 2014 [27] | | 66.0% |  | | < 2 cm |  |
| Chou 2014 [27] | | 78.0% |  | | Lesions, 1– cm |  |
| **Mean** | **72.0%** | | **78.9%*** | | **Specificity estimated based on the sensitivity and specificity difference for overall HCC* |  |
|  |  | |  | |  |  |
| **GD-EOB-DTPA-MRI subsequent imaging** | | | | |  |  |
| Chen 2013 [25] | | 87.0% | 93.0% | | MRI with liver-specific contrast agents; size <20 mm | Sensitivity: 15; specificity: 5 |
| **Mean** | **87.0%** | | **93.0%** | |  |  |
