# Supplementary material for: Health economic assessment of Gd-EOB-DTPA MRI versus ECCM-MRI and multi-detector CT for diagnosis of hepatocellular carcinoma in China
Source: PLoS One. 2018 Jan 11;13(1):e0191095. doi: 10.1371/journal.pone.0191095 (PMC5764342; doi:10.1371/journal.pone.0191095)
Supplement: S3 Table — (DOCX) [file pone.0191095.s004.docx]

**S3 Table Costs of diagnostic and treatment procedures in China**

| **Diagnostics and treatments** | **Cost (¥)** |
| --- | --- |
| **Diagnostics** |  |
| MDCT | ¥1,528 |
| ECCM-MRI | ¥1,558 |
| Gd-EOB-DTPA-MRI | ¥2,549 |
| FNA | ¥1,981 |
| **Treatments** |  |
| Excision | ¥60,832 |
| TACE | ¥25,682 |
| Radiofrequency ablation | ¥31,750 |
| Microwave ablation | ¥29,500 |
| Liver transplant | ¥261,032 |

ECCM-MRI, extracellular contrast media–enhanced MRI; Gd-EOB-DTPA-MRI, Gd-EOB-DTPA–enhanced magnetic resonance imaging; MDCT, multidetector computed tomography; TACE, transcatheter arterial chemoembolization
