# Supplementary material for: Health economic assessment of Gd-EOB-DTPA MRI versus ECCM-MRI and multi-detector CT for diagnosis of hepatocellular carcinoma in China
Source: PLoS One. 2018 Jan 11;13(1):e0191095. doi: 10.1371/journal.pone.0191095 (PMC5764342; doi:10.1371/journal.pone.0191095)
Supplement: S4 Table — (DOCX) [file pone.0191095.s005.docx]

**S4 Table Final true prevalence**

| **Expert No.** | **Department** | **City** | **Estimated number of confirmed HCC cases out of 100 suspected patients at high risk** | | |
| --- | --- | --- | --- | --- | --- |
|  |  |  | **Lowest** | **Most likely** | **Highest** |
| 1 | Liver diseases | Beijing | 20 | 40 | 40 |
| 2 | Liver diseases | Beijing | 60 | 70 | 80 |
| 3 | Liver diseases | Beijing | 2 | 40 | 80 |
| 4 | Liver diseases | Beijing | 10 | 30 | 50 |
| 5 | Liver diseases | Shanghai | 20 | 40 | 80 |
| 6 | Liver diseases | Shanghai | 20 | 40 | 80 |
| 7 | Liver diseases | Guangzhou | 20 | 30 | 60 |
| 8 | Liver diseases | Shenzhen | 20 | 40 | 70 |
| 9 | Infection | Guangzhou | 10 | 35 | 50 |
| 10 | Infection | Guangzhou | 20 | 50 | 80 |
| 11 | Infection | Nanning | 10 | 45 | 80 |
| 12 | Infection | Chongqing | 20 | 40 | 70 |
| 13 | Infection | Zhengzhou | 10 | 40 | 80 |
| 14 | Infection | Suzhou | 5 | 40 | 80 |
| 15 | Radiology | Beijing | 30 | 50 | 80 |
| 16 | Radiology | Beijing | 25 | 55 | 60 |
| 17 | Radiology | Shanghai | 30 | 50 | 70 |
| 18 | Radiology | Shanghai | 20 | 40 | 70 |
| 19 | Radiology | Guangzhou | 20 | 50 | 70 |
| 20 | Radiology | Guangzhou | 50 | 60 | 70 |
| 21 | Radiology | Guangzhou | 40 | 50 | 60 |
| 22 | Radiology | Shenzhen | 30 | 50 | 80 |
| 23 | Radiology | Nanjing | 40 | 60 | 80 |
| 24 | Radiology | Chongqing | 30 | 55 | 70 |
| 25 | Radiology | Zhengzhou | 40 | 55 | 80 |
| 26 | Radiology | Suzhou | 35 | 50 | 70 |
| **Total** | | | **25** | **46** | **71** |
|  |  |  | **47** | | |

Final HCC true prevalence was calculated as the average rate of the lowest, most likely, and highest estimated rates using the Delphi approach.
