# Supplementary material for: Health economic assessment of Gd-EOB-DTPA MRI versus ECCM-MRI and multi-detector CT for diagnosis of hepatocellular carcinoma in China
Source: PLoS One. 2018 Jan 11;13(1):e0191095. doi: 10.1371/journal.pone.0191095 (PMC5764342; doi:10.1371/journal.pone.0191095)
Supplement: S5 Table — (DOCX) [file pone.0191095.s006.docx]

**S5 Table Weighted treatment costs determined by distributions of tumor groups and treatment procedures**

| **Tumor group** | **Excision** | **TACE** | **RFA** | **MA** | **LT** | **Total** | **Weighted^*^** | **Distr.^#^** | **Weighted^**^** |
| --- | --- | --- | --- | --- | --- | --- | --- | --- | --- |
| 1 Tumor <5 cm | 50.2% | 25.9% | 22.2% | 1.0% | 0.7% | 100% | ¥46,364 | 47.1% | ¥21,826 |
| 1 Tumor ≥5 cm | 76.2% | 16.9% | 3.4% | 0.8% | 2.8% | 100% | ¥59,181 | 25.3% | ¥14,973 |
| 2–3 Tumors, all tumors ≤3 cm | 41.8% | 29.8% | 19.1% | 1.0% | 8.3% | 100% | ¥60,980 | 8.2% | ¥5,000 |
| 2–3 Tumors, ≥1 tumor >3 cm | 31.5% | 22.3% | 31.9% | 4.3% | 10.0% | 100% | ¥62,389 | 6.4% | ¥3,993 |
| >3 Tumors | 21.5% | 31.1% | 18.5% | 3.8% | 25.2% | 100% | ¥93,712 | 13.0% | ¥12,206 |
| **Total** | | | | | | | **—** | 100% | ¥57,998 |

LT, Liver transplant; MA, microwave ablation; RFA, radiofrequency ablation; TACE, Transcatheter arterial chemoembolization.

*Weighted by distribution of treatment procedures within each tumor group.

**Weighted by distribution of tumor groups.

^#^Distribution of tumor groups in terms of tumor number and size among patients with confirmed HCC diagnosis
