# Supplementary material for: Health economic assessment of Gd-EOB-DTPA MRI versus ECCM-MRI and multi-detector CT for diagnosis of hepatocellular carcinoma in China
Source: PLoS One. 2018 Jan 11;13(1):e0191095. doi: 10.1371/journal.pone.0191095 (PMC5764342; doi:10.1371/journal.pone.0191095)
Supplement: S6 Table — (DOCX) [file pone.0191095.s007.docx]

**S6 Table One-way sensitivity analysis of cost differences between Gd-EOB-DTPA-MRI and MDCT**

| Scenario | Cost difference  (EOB-MRI vs. MDCT) |
| --- | --- |
| Base case | -¥442 |
| Sensitivity of initial MDCT increased to 81.0% (base case: 73.4%) | -¥1,393 |
| Sensitivity of initial MDCT decreased to 66.0% (base case: 73.4%) | ¥471 |
| True HCC prevalence among patients with suspected HCC increased by 10% (base case: 47.0%) | ¥81 |
| True HCC prevalence among patients with suspected HCC decreased by 10% (base case: 47.0%) | -¥966 |
| Probability of needing further diagnostics among negatives at initial MDCT increased by 10% (base case: 61.4%) | -¥959 |
| Probability of needing further diagnostics among negatives at initial MDCT decreased by 10% (base case: 61.4%) | ¥74 |
| Specificity of initial MDCT increased to 93.0% (base case: 91.4%) | ¥9 |
| Specificity of initial MDCT decreased to 89.8% (base case: 91.4%) | -¥880 |
| Sensitivity of initial EOB-MRI increased to 95.0% (base case: 92.3%) | -¥7 |
| Sensitivity of initial EOB-MRI decreased to 91.0% (base case: 92.3%) | -¥640 |
| Unit cost of EOB-MRI increased by 10% (base case: ¥2,549) | -¥256 |
| Unit cost of EOB-MRI decreased by 10% (base case: ¥2,549) | -¥628 |
| Specificity of initial EOB-MRI increased to 96.0% (base case: 95.3%) | -¥670 |
| Specificity of initial EOB-MRI decreased to 95.0% (base case: 95.3%) | -¥336 |
| Treatment cost per patient with HCC increased by 10% (base case: ¥57,998) | -¥515 |
| Treatment cost per patient with HCC decreased by 10% (base case: ¥57,998) | -¥370 |
| Inclusion of treatment costs for false negatives as ¥57,998 per patient (base case: ¥0) | -¥6,235 |

MDCT, multidetector computed tomography; ECCM-MRI, extracellular contrast media–enhanced MRI; EOB-MRI/Gd-EOB-DTPA-MRI, Gd-EOB-DTPA–enhanced magnetic resonance imaging; HCC, hepatocellular carcinoma; HCC, hepatocellular carcinoma.
