# Supplementary material for: Health economic assessment of Gd-EOB-DTPA MRI versus ECCM-MRI and multi-detector CT for diagnosis of hepatocellular carcinoma in China
Source: PLoS One. 2018 Jan 11;13(1):e0191095. doi: 10.1371/journal.pone.0191095 (PMC5764342; doi:10.1371/journal.pone.0191095)
Supplement: S7 Table — (DOCX) [file pone.0191095.s008.docx]

**S7 Table One-way sensitivity analysis of cost differences between Gd-EOB-DTPA-MRI and ECCM-MRI**

| Scenario | Cost difference  (EOB-MRI vs. ECCM-MRI) |
| --- | --- |
| Base case | -¥1,104 |
| True HCC prevalence among patients with suspected HCC increased by 10% (base case: 47.0%) | -¥630 |
| True HCC prevalence among patients with suspected HCC decreased by 10% (base case: 47.0%) | -¥1,579 |
| Specificity of initial EOB-MRI increased to 96.0% (base case: 95.3%) | -¥1,332 |
| Specificity of initial EOB-MRI decreased to 95.0% (base case: 95.3%) | -¥1,028 |
| Probability of needing further diagnostics among negatives at initial ECCM-MRI increased by 10% (base case: 51.6%) | -¥1,411 |
| Probability of needing further diagnostics among negatives at initial ECCM-MRI decreased by 10% (base case: 51.6%) | -¥798 |
| Sensitivity of initial ECCM-MRI increased to 81.0% (base case: 79.7%) | -¥1,285 |
| Sensitivity of initial ECCM-MRI decreased to 78.0% (base case: 79.7%) | -¥879 |
| Treatment cost per patient with HCC increased by 10% (base case: ¥57,998) | -¥1,262 |
| Treatment cost per patient with HCC decreased by 10% (base case: ¥57,998) | -¥946 |
| Unit cost of EOB-MRI increased by 10% (base case: ¥2,549) | -¥908 |
| Unit cost of EOB-MRI decreased by 10% (base case: ¥2,549) | -¥1,301 |
| Sensitivity of initial EOB-MRI increased to 95.0% (base case: 92.3%) | -¥669 |
| Sensitivity of initial EOB-MRI decreased to 91.0% (base case: 92.3%) | -¥1,302 |
| Probability of needing further diagnostics among negatives at initial EOB-MRI increased by 10% (base case: 31.8%) | -¥1,037 |
| Probability of needing further diagnostics among negatives at initial EOB-MRI decreased by 10% (base case: 31.8%) | -¥1,172 |
| Inclusion of treatment costs for false negatives as ¥57,998 per patient (base case: ¥0) | -¥4,825 |

MDCT, multidetector computed tomography; ECCM-MRI, extracellular contrast media–enhanced MRI; EOB-MRI/Gd-EOB-DTPA-MRI, Gd-EOB-DTPA–enhanced magnetic resonance imaging; HCC, hepatocellular carcinoma; HCC, hepatocellular carcinoma.
